# *Sicuophora* (Syn. *Wichtermania*) *multigranularis* from *Quasipaa spinosa* (Anura): morphological and molecular study, with emphasis on validity of *Sicuophora* (Armophorea, Clevelandellida)

**DOI:** 10.1051/parasite/2018035

**Published:** 2018-07-27

**Authors:** Can Li, Weishan Zhao, Dong Zhang, Runqiu Wang, Guitang Wang, Hong Zou, Wenxiang Li, Shangong Wu, Ming Li

**Affiliations:** 1 Key Laboratory of Aquaculture Disease Control, Ministry of Agriculture, and State Key Laboratory of Freshwater Ecology and Biotechnology, Institute of Hydrobiology, Chinese Academy of Sciences Wuhan 430072 China; 2 Hubei Key Laboratory of Animal Nutrition and Feed Science, Wuhan Polytechnic University Wuhan 430023 China

**Keywords:** Clevelandellida, Endocommensal, Phylogenetic analysis, Protargol, Scanning electron microscopy, SSU-rRNA

## Abstract

Morphological studies of *Sicuophora* (Syn. *Wichtermania*) *multigranularis* Xiao et al., 2002, from the rectum of the frog, *Quasipaa spinosa*, performed using silver impregnation and scanning electron microscopy, confirmed the following newly recognized features: (1) only one apical suture on the right surface; (2) two naked regions at the posterior end of both the left and the right side of the body. Phylogenetic analysis based on the SSU-rRNA gene showed that *S. multigranularis* is a sister to a clade comprising all other Clevelandellida, strongly supporting the validity of the genus *Sicuophora*. This is also the first molecular data obtained for the genus *Sicuophora*. Because of the lack of molecular data, it will be necessary to obtain more genetic data from the family Sicuophoridae to discuss the question of the taxonomic status of the genus *Sicuophora*.

## Abstract (fr)

Des études morphologiques de *Sicuophora* (Syn. *Wichtermania*) *multigranularis* Xiao et al., 2002, du rectum de la grenouille *Quasipaa spinosa*, réalisées en utilisant imprégnation argentique et microscopie électronique à balayage, ont confirmé les caractéristiques suivantes, nouvellement reconnues: (1) une seule suture apicale sur la surface droite; (2) deux régions nues à l’extrémité postérieure du côté gauche et du côté droit du corps. L’analyse phylogénétique basée sur le gène SSU-rRNA a montré que *S. multigranularis* était le groupe-frère d’un clade comprenant tous les autres Clevelandellida, ce qui confirme fortement la validité du genre *Sicuophora*. Ceci est aussi la première donnée moléculaire du genre *Sicuophora*. En raison du manque de données moléculaires, il sera nécessaire d’obtenir plus de données génétiques de la famille Sicuophoridae pour discuter de la question du statut taxonomique du genre *Sicuophora*.

## Introduction

Clevelandellid ciliates (Armophorea: Clevelandellida) are a large group of anaerobic ciliates, mainly inhabiting the digestive system of various hosts from oligochaetes, insects (cockroaches), myriapods (centipedes, millipedes) and molluscs (shipworms) to fishes, amphibians (frogs and toads), and reptiles (lizards) [9, 15].

Concerning the classification of clevelandellids, several revisions have been made during the past 50 years. Earl (1972) created a superfamily, Plagiotomoidea, for this group comprising two families Plagiotomidae Bütschli, 1887 and Clevelandellidae Kidder, 1938 [7]. De Puytorac & Grain (1976) created the suborder Clevelandellina, which was accepted by Corliss (1977, 1979) and divided it into five families: Nyctotheridae Amaro, 1972, Sicuophoridae Amaro, 1972, Clevelandellidae Kidder, 1938, Inferostomatidae Ky, 1971, and Nathellidae Singh, 1953 [3, 4, 19]. Lynn (2008) elevated the suborder to ordinal rank Clevelandellida de Puytorac & Grain, 1976, and included it in the class Armophorea Lynn, 2004 [14, 15]. He retained the four families Nyctotheridae, Sicuophoridae, Clevelandellidae and Inferostomatidae, but considered the fifth family, Nathellidae a synonym of Inferostomatidae and introduced the family Neonyctotheridae Affa’a, 1987.

Earl (1972) transferred *Nyctotherus cheni* and *N. skalii* from the genus *Nyctotherus* into a new genus *Wichtermania*. He named the new genus in honor of Dr. Ralph Wichterman who discovered *Nyctotherus cheni* in 1934 [7]. In addition to these two originally included *Wichtermania* species, there are six other congeners, all discovered in China, including *W. multigranularis*, *W. oviformis*, *W. vesiformis*, *W. reticulatis*, *W. granuliformis*, and *W. obliquoides* [24]. However, Corliss (1979) synonymized the genus *Wichtermania* with the genus *Sicuophora*, and Lynn (2008) has followed this decision and assigned it to the family Sicuophoridae as having “inferior concave surface in part or whole as ‘sucker’ with supporting polysaccharide skeletal elements” [4, 15]. We agree with this revision and use the generic name *Sicuophora* throughout the paper. Morphological descriptions of *Sicuophora* species are, however, rather incomplete and some important taxonomic features need careful re-examination. Currently, there are no molecular data from *Sicuophora* species. Here, we chose *S. multigranularis* as a representative of the genus in order to re-evaluate its morphologic features using silver impregnation and scanning electron microscopy (SEM). We provide the first SSU rRNA gene sequence for the genus *Sicuophora*, and assess its systematic position by phylogenetic analyses.

## Material and methods

### Specimen collection, isolation and identification

The examined dicroglossid frogs, *Quasipaa spinosa*, were captured in Lishui city (27°25′–28°57′N; 118°41′–120°26′E), Zhejiang Province, China in May to August 2017. We obtained the permits allowing us to capture and sacrifice these specimens. The frogs were transported live to the laboratory for further examination. All frog specimens were anesthetized by inhalation of ether and then dissected as soon as possible. The rectum, intestines and duodenum were isolated in separate Petri dishes and examined with the aid of a stereoscopic microscope Stemi SV6/AxioCam MRc5 (Zeiss, Oberkochen, Germany). The living ciliates were collected with Pasteur micropipettes and washed twice in 0.65% physiological saline.

For identification, specimens were smeared on microscope slides and stained with protargol [23]. For measurements, we used freshly killed ciliates (in 5% formalin) without coverslips. The specimens were observed, measured at 200× or 400× magnification, and photographed using Axioplan 2 imaging and Axiophot 2 (Zeiss, Oberkochen, Germany). All measurements are in micrometers.

For scanning electron microscopy (SEM), the washed specimens were fixed in 2.5% glutaraldehyde in 0.2 M phosphate buffered saline (PBS, pH 7.4) on a clean glass slide (1 cm × 1 cm). The glass slides were pre-treated with 0.1% poly-L-Lysine and completely air-dried at room temperature. After washing three times with PBS, cells were post-fixed in 1% osmium tetroxide at 4 °C for 1 h, followed by serial dehydration in acetone and critical point drying using an HCP-2 critical point dryer (Hitachi Science Systems, Ibaraki, Japan). Then, the glass slides were mounted on aluminum stubs using double-sided adhesive tape and sputter-coated with a thin layer of gold in an IB-3 ion coater (Eiko Engineering, Ibaraki, Japan) before observation and photographing with a Quanta 200 SEM (FEI, Amsterdam, Netherlands).

### Extraction of genomic DNA and SSU-rRNA gene sequencing

About 100 ciliates were harvested from *Quasipaa spinosa*, suspended in lysis buffer (10 mM Tris-HCl, pH 8.0; 1 M EDTA, pH 8.0; 0.5% sodium dodecyl sulfate; 60 μg/mL proteinase K), and incubated at 55 °C for 12–20 h. The DNA was extracted using a standard phenol/chloroform method, precipitated with ethanol, and resuspended in TE buffer (10 mM Tris-HCl, pH 8.0; 1 mM EDTA, pH 8.0) [21].

Polymerase chain reaction (PCR) amplifications were carried out in 25 μL volume reactions containing 1 μL of template DNA, 1 μM of both forward and reverse primers (F: 5′-AACCTGGTTGATCCTGCCAGT-3′; R: 5′-TGATCCTTCTGCAGGTTCACCTAC-3′) [17], 0.2 mM dNTP, 2 mM MgCl_2_, and 1 U of Taq DNA polymerase (Fermentas, Foster City, CA, USA). An EDC-810 DNA Engine (EastWin Bio., Co., Ltd, Beijing, China) was used to control the cycling conditions: five cycles of denaturation for 1 min at 94 °C, primer annealing for 2 min at 56 °C and extension for 2 min at 72 °C, followed by 35 cycles with the annealing temperature increased to 62 °C.

The PCR products were isolated using 1% agarose gel electrophoresis and purified using the Agarose Gel DNA Purification Kit Ver. 2.0 (TaKaRa Biotechnology, Dalian, China). The amplified fragments were cloned into a pMD-18T vector (TaKaRa Biotechnology) and four positive clones were chosen for sequencing in both directions using M13 forward and reverse primers on an ABI PRISM^®^ 3730 DNA Sequencer (Applied Biosystems, Foster City, CA, USA).

### Sequence availability and phylogenetic analyses

To reveal the systematic position of *Sicuophora multigranularis*, 46 armophorean SSU-rRNA gene sequences were retrieved from the GenBank/EMBL databases (for accession numbers, see Fig. 5); the sequence of *Balantidium ctenopharyngodoni* was used as the outgroup.

The secondary structure-based SSU-rRNA gene sequence alignment of the phylum Ciliophora downloaded from the SILVA rRNA database (https://www.arb-silva.de/) was used as the “seed” alignment to build a profile hidden Markov model (HMM), using the HMMER Package, version 2.3.2. The resulting HMM profile was then used to create an alignment of the 47 sequences using Hmmalign within the package. The resulting alignment was further modified manually using MEGA6 [22] and 1722 characters were finally used for subsequent phylogenetic analyses.

Phylogenetic trees were constructed by the maximum-likelihood (ML) method and the Bayesian (BI) method, as implemented in PhyML 3.0 [8] and MrBayes 3.2 [20], respectively. The program jModelTest 2 [6] selected the GTR+G+I as the best model with AIC criterion, which was then used for both Bayesian and ML inference. For ML analysis, a BioNJ starting tree was used for tree searching, and data were bootstrap resampled 1000 times. A Markov Chain Monte Carlo (MCMC) algorithm was used in BI analysis, with four chains running for 1,000,000 generations, with sampling every 1000th tree. Relative burn-in was set to 25%, and otherwise default settings were used.

## Results

Numerous *S. multigranularis* were found in the recta of the examined frogs.

### Morphological description based on the Lishui population (Figs. 1–4; Table 1)

Slides 2017W013-020 of protargol-stained specimens have been deposited at the Institute of Hydrobiology, Chinese Academy of Sciences, Wuhan, China.

**Figure 1.**
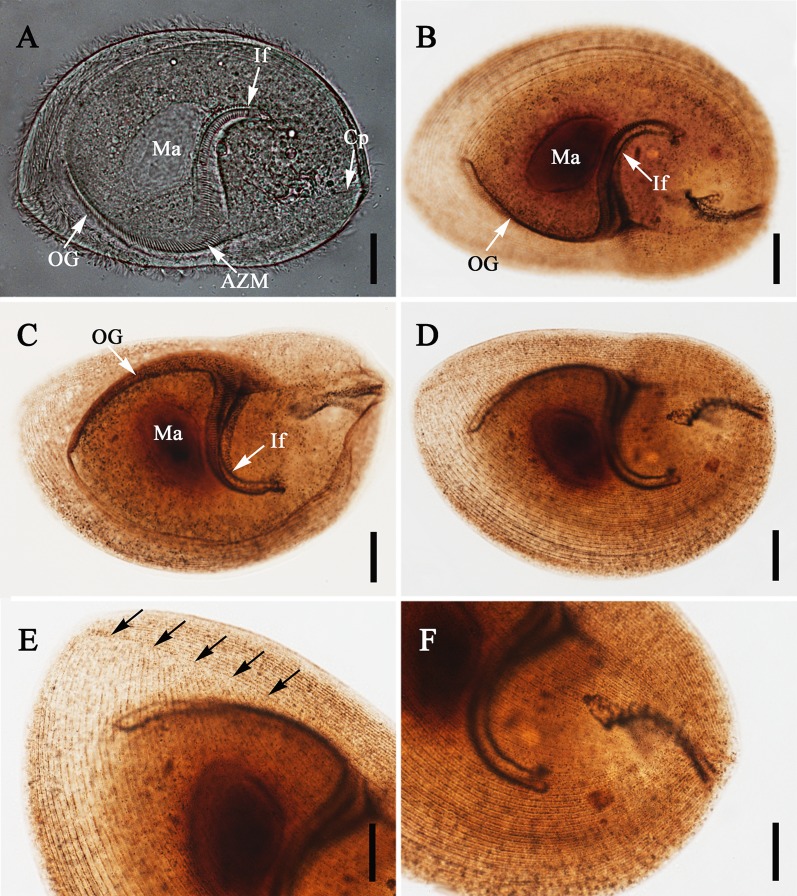
Light microscope images of *Sicuophora multigranularis*. (A) Left surface view of a living, representative specimen, to show the macronucleus (Ma), oral groove (OG), adoral zone of membranelles (AZM), infundibulum (If), and cytoproct (Cp). Scale bar = 30 μm. (B)–(F) Specimens stained with protargol. **(**B)–(C) Left and right side view showing the oral groove (OG), infundibulum (If), and macronucleus (Ma). Scale bar = 30 μm. (D) Right side overview, showing the somatic kinety pattern. Scale bar =30 μm. (E) Detail of anterior body region, showing the apical, right suture (arrows). Scale bar = 20 μm. (F) Detail of posterior body region, showing the course of right side ciliary rows. Scale bar = 20 μm.

**Table 1.** Biometric data (in μm) on *Sicuophora multigranularis* and comparison with former report.

Species	Locality	Parameter										References
			BL (L)	BW (L)	BL (R)	BW (R)	OgL	IfL	IfW	MaL	MaW	
*S. multigranularis*	Zhejiang, China	$\bar{X}$	171.9	105.0	198.5	132.9	102.2	112.1	20.1	44.0	29.9	Present study
		*M*	168.7	102.4	196.5	134.1	104.9	108.9	20.2	44.5	30.2	
		Max	202.0	126.7	228.2	158.0	120.0	133.5	29.8	53.5	38.6	
		Min	154.6	85.6	180.8	115.6	87.5	94.7	14.7	34.8	24.5	
		*SD*	13.3	10.8	13.7	10.8	8.7	11.8	3.6	5.1	3.8	
		CV (%)	7.8	10.3	6.9	8.1	8.5	10.5	17.7	11.5	12.8	
		*N*	20	20	20	20	20	20	20	20	20	
*W. multigranularis*	Hubei, Guangxi, China	$\bar{X}$	189.0	127.8	209.0	140.9	90.7	120.0	23.8	44.9	31.7	Xiao et al. [24]
		Max	224.1	143.1	224.0	151.2	105.3	127.6	29.7	51.3	37.8	
		Min	164.7	108.0	191.7	124.2	83.7	120.6	21.6	35.1	27.0	

Measurements in µm; $\bar{X}$ = arithmetic mean, *M* = median, *Max* = Maximum, *Min* = Minimum, *SD* = standard deviation, *CV* = coefficient of variation, *N* = number of individuals investigated; *BL(L)* = Body length of left surface, *BW(L)* = Body width of left surface, *BL(R)* = Body length of right surface, *BW(R)* = Body width of right surface, *OgL* = Oral groove length, *IfL* = Infundibulum length, *IfW* = Infundibulum width, *MaL* = Macronucleus length, *MaW* = Macronucleus width.

**Figure 2.**
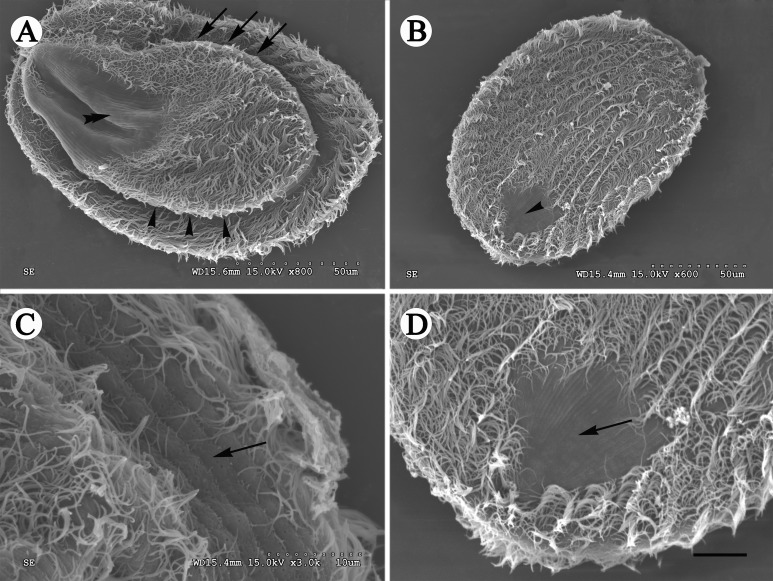
SEM images of *Sicuophora multigranularis*. (A) Left side view showing the oral groove (arrows) and its convex left side as well as the naked region without cilia at the posterior body region (double arrowheads), and two very different sides. Scale bar = 50 μm. (B) Right side view, showing the densely arranged ciliary rows and the glabrous region without cilia in the posterior body region. Scale bar = 50 μm. (C) Detail of specimen shown in Fig. 2A. Note the sparely arranged cilia on the cell margin. Scale bar = 10 μm. (D) Detail of specimen shown in Fig. 2B. There is a conspicuous naked area on the right side near the posterior body end. Scale bar = 10 μm.

**Figure 3.**
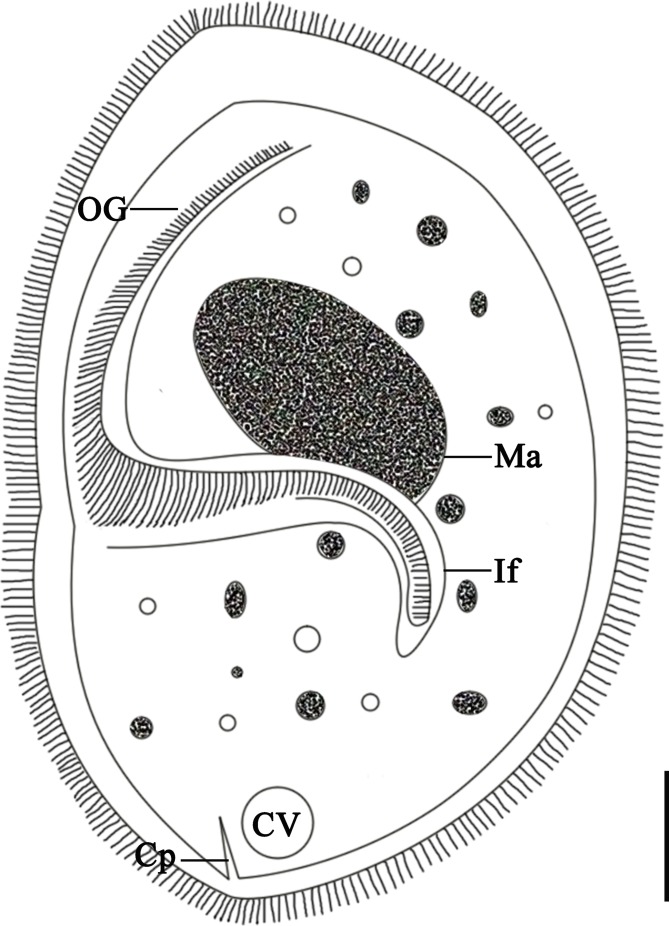
Schematic drawing of *Sicuophora multigranularis*, showing the general form and structures of the left side: the oral groove (OG), infundibulum (If), macronucleus (Ma), contractile vacuole (CV) and cytoproct (Cp). Scale bar = 30 μm.

Organism oval or elliptical shaped, pointed at anterior end and rounded at posterior end, and densely ciliated (Figs. 1A–1D, 2A, 2B, and 3). Two sides of the cell significantly different (Figs. 1A, 2A, and 3). Left surface irregularly convex, slightly thicker at anterior end than posterior end, right surface flat or slightly concave (Figs. 2A and 2B). Right side large, while left side comparatively small, leaving prominent wide margin (about 15–20 μm in width) (Fig. 2A). Left side body length 154.6–202.0 μm ($\bar{X}$ = 171.9 μm; *n* = 20) and width 85.6–126.7 μm ($\bar{X}$ = 105.0 μm; *n* = 20). Right side body length 180.8–228.2 μm ($\bar{X}$ = 198.5 μm; *n* = 20) and width 115.6–158.0 μm ($\bar{X}$ = 132.9 μm; *n* = 20).

Oral groove starts at anterior extremity of left side, extends to near 1/2–2/3 of body length of left side ($\bar{X}$ = 102.2 μm, 87.5–120.0 μm; *n* = 20) (Figs. 1A–1C and 3). Adoral zone of membranelles (AZM) originates at anterior end of body, extends toward posterior end of infundibulum. Infundibulum 94.7–133.5 μm ($\bar{X}$ = 112.1 μm; *n* = 20) in length, 14.7–29.8 μm ($\bar{X}$ = 20.1 μm; *n* = 20) in width, J-shaped, extending into posterior part of body forming an angle of about 60–80° to its longitudinal axis (Figs. 1A–1C and 3). The cytoproct in form of a short canal, emptying at the posterior body end (Figs. 1A and 3).

**Figure 4.**
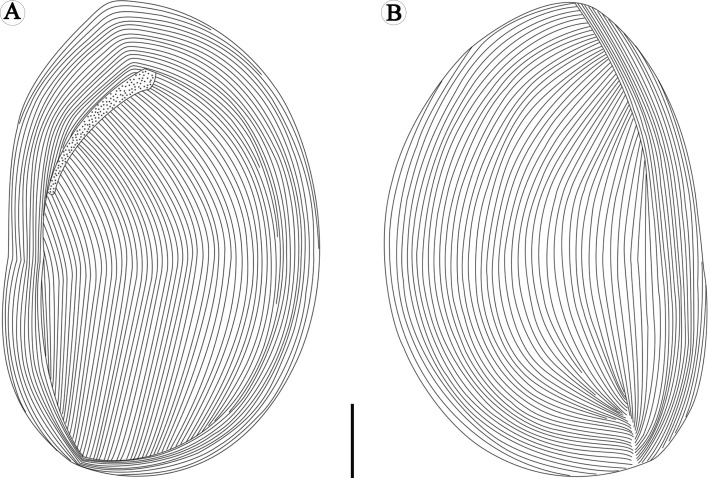
Schematic drawings of *Sicuophora multigranularis*, showing ciliary rows on the left side, where they do not form any suture (A) and ciliary rows on the right side, where they form an apical suture (B). Scale bar = 30 μm.

Ciliary rows of left surface start from lateral border of oral groove, extend to posterior end as arc without suture, cilia clearly observed on flange which encircles the body, glabrous region having no cilia at posterior end (Figs. 2A and 4A). Distinct apical suture on anterior end of right surface, ciliary rows on exterior of left side originating from suture converge, in arciform fashion to posterior part, several parallel ciliary rows on right side, also with naked region at posterior end (Figs. 1D–1F, 2B, 2D and 4B). Several sparse cilia scattered on the margin (Fig. 2C).

Macronucleus broadly ellipsoidal shaped, 34.8–53.5 μm ($\bar{X}$ = 44.0 μm; *n* = 20) in length, 24.5–38.6 μm ($\bar{X}$ = 29.9 μm; *n* = 20) in width. Lies in anterior half of body above infundibulum (Figs. 1A–1C). Micronucleus not observed. Cytoplasm colorless, containing many different-sized granules as well as numerous small and large vacuoles (Figs. 1A and 3). Contractile vacuole situated at posterior end of body (Fig. 3).

### Characterization of the SSU-rRNA gene and phylogenetic analyses

The sequences from the four clones analyzed were identical. The complete SSU-rRNA gene sequence is 1722 nucleotides in length and its GC content is 45.24%. This sequence has been deposited in the GenBank/EMBL/DDBJ database under the accession number MH301103.

**Figure 5.**
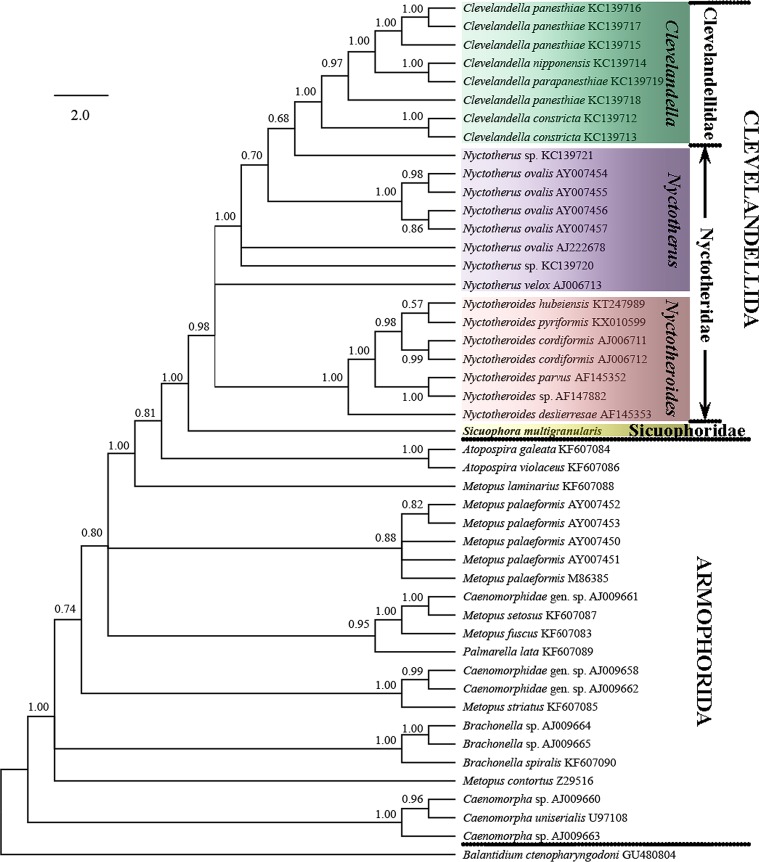
SSU rRNA gene phylogenetic tree of ciliates from the class Armophorea, inferred by the Bayesian analysis. The tree has been rooted using the sequence of *Balantidium ctenopharyngodoni*. The posterior probability values are shown next to the branches. The analysis involved 47 nucleotide sequences. The tree is drawn to scale, with branch lengths measured in the number of substitutions per site.

**Figure 6.**
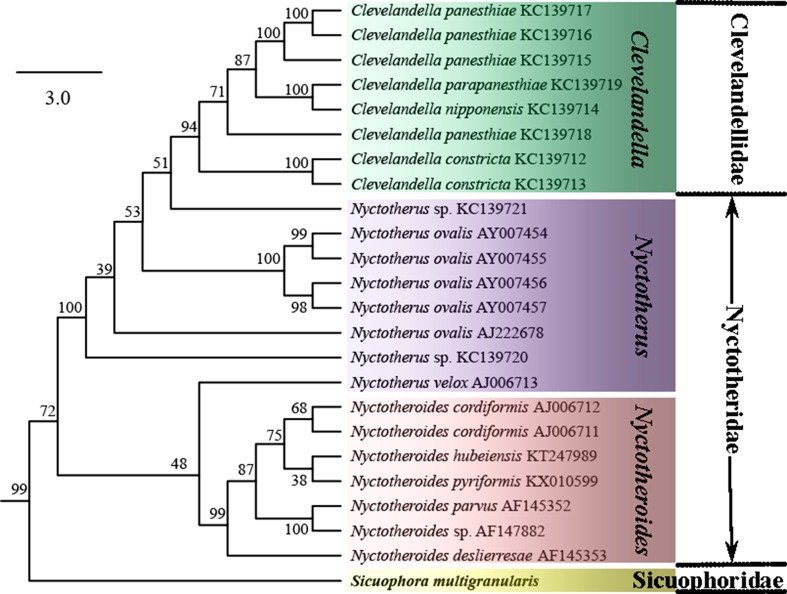
Subtree extracted from the SSU rRNA gene phylogenetic tree inferred by the Maximum Likelihood method, showing only phylogenetic relationships among members of the order Clevelandellida. The bootstrap values are shown next to the branches. The subtree is drawn to scale, with branch lengths measured in the number of substitutions per site.

The tree topologies constructed by the ML and BI methods were similar, especially the branching pattern within the Clevelandellida clade. In the phylogenetic trees, *S. multigranularis* was sister to a clade comprising all clevelandellids, as a sister clade to Clevelandellidae + Nyctotheridae (99% [ML], 1.00 [BI]) (Figs. 5 and 6). The order Clevelandellida (including *Clevelandella* [family Clevelandellidae], *Nyctotherus* and *Nyctotheroides* [family Nyctotheridae], *Sicuophora* [family Sicuophoridae]) is fully supported as a monophyletic clade. The genus *Nyctotheroides* was depicted as monophyletic (99% [ML], 1.00 [BI]), while the genus *Nyctotherus* was shown as paraphyletic, encompassing members of the genus *Clevelandella*. Thus, the family Nyctotheridae (*Nyctotherus* and *Nyctotheroides*) was revealed to be paraphyletic, while the family Clevelandellidae (*Clevelandella*) was supported as monophyletic (94% [ML], 1.00 [BI]).

## Discussion

*Sicuophora* (Syn. *Wichtermania*) *multigranularis*, first discovered in the rectum of *Quasipaa spinosa*, was described and named by Xiao et al. (2002) [24]. The morphologic features of our specimens fully correspond to those in the original description. *Sicuophora multigranularis* shows quite strict host-specificity to *Q. spinosa* and inhabits only the rectum. Therefore, we confirm that it is scientifically sound to identify our specimens as the same species recorded by Xiao et al. (2002) [24].

With regard to its morphological features, some revisions need to be pointed out here based upon our observations: (1) only one apical suture, on the right surface of *S. multigranularis*, was observed. We did not observe what was described by Xiao et al. (2002) as a “λ” shaped suture line on the left surface of the organism, although we carefully checked our living, protargol and silver nitrate stained specimens. We speculated that the so-called “λ“ shaped suture line on the left side of the ciliate was an artifact occurring from the junction of two sides when the specimens were smeared and dried on cover slips for silver-staining (see Fig. 4A). In addition, according to our SEM observations, the two surfaces of the organism are not identical – left (dorsal) side narrower and convex, right (ventral) broader and flat. This fact can cause an optical illusion of a “λ” suture line on the left side when observed using light microscopy. Therefore, this feature is likely an artifact. (2) There are two naked regions at the posterior end of both the left and the right sides of the body. This is inconsistent with the description of Xiao et al. (2002), since they mentioned only one naked region on the left side. In fact, the naked region is hard to distinguish with light microscopy, but is well identified in SEM images.

*Nyctotherus*, *Nyctotheroides*, and *Sicuophora* share an oval or elliptical body with convex left side and flat or slightly concave right side. Their macronucleus is roughly oblong or sausage-shaped, lies in the anterior half of the body and above the infundibulum. The cytoproct is distinct and slit-like and the single contractile vacuole is situated at the posterior end. The major differences between *Nyctotherus* and *Nyctotheroides* are the number and location of the suture line and the hosts [2]. The genus *Sicuophora* was separated from the genus *Nyctotheroides* based on having a conspicuous “sucker” on its anterolateral right side [7]. Considering the features of kinetal suture patterns, morphological criteria for distinguishing *Nyctotheroides* from *Nyctotherus*, according to our previous studies are as follows: *N. huibeiensis* (Li et al. 1998) has only one apical suture line on the left side, while *N. pyriformis* (Nie, 1932, Li et al. 2002, Li et al. 2017) possesses a caudal suture on the left side and an apical suture on the right side [10–13, 18]. Another six *Nyctotheroides* species, first reported in China, including *N. liuzhouensis*, *N. ellipticus*, *N. luojiaensis*, *N. pigmentosae*, *N. rhacophori*, and *N. bufoides*, were however described as having no suture line [10]. As to *Sicuophora* (Syn. *Wichtermania*), there were another five species (aforementioned) all first discovered in China, reported to have a “λ“ suture line on both sides, while only one apical suture line was observed on the right side of *S. multigranularis*. It seems that the number and location of the suture lines cannot distinguish the three aforementioned genera unambiguously, and hence this feature should not be considered as an applicable generic taxonomic criterion.

The branching pattern of the ML and BI trees was almost identical for the order Clevelandellida, while some differences (data not shown) were found in the order Armophorida. *S*. *multigranularis* was consistently placed as a sister taxon of all other members of the order Clevelandellida, which strongly supports the distinctness of the genus *Sicuophora*. Thus, *Sicuophora* was well separated from *Nyctotherus* spp. and *Nyctotheroides* spp. Taking into account genetic and morphological data, it is reasonable for us to confirm the validity of genus *Sicuophora*. However, re-sampling of the aforementioned five Chinese *Sicuophora* (Syn. *Wichtermania*) species and careful investigation of their morphology and SSU rRNA gene sequences is needed to determine their phylogenetic position and to clarify monophyly of the genus *Sicuophora*.

Both Corliss (1979) and Lynn (2008) distinguish the taxa assigned to the family Sicuophoridae as having “inferior concave surface in part or whole as ‘sucker’ with supporting polysaccharide skeletal elements” [4, 15]. This feature separates these genera from those assigned to the families Nyctotheridae, Neonyctotheridae, and Clevelandellidae. The *Wichtermania* species redescribed by these authors has a conspicuous “sucker” on its inferior surface and so can be unambiguously assigned to the family Sicuophoridae. SSU rRNA gene sequences were obtained for the first time for the genus *Sicuophora*, which presumably belongs to the family Sicuophoridae. Since no other sicuophorid genus has been sequenced so far, it is not possible to analyze the exact affiliation of the genus *Sicuophora*, and further molecular data from the family Sicuophoridae are needed. As concerns the phylogenetic relationships within the class Armophorea, the order Clevelandellida seems to have been derived from free-living members of the paraphyletic order Armophorida. Interrelationships within the order Clevelandellida are in accordance with former studies [1, 5, 12, 13, 16]. Specifically, endocommensals in anurans (*Nyctotheroides*) are separated from those in arthropods (*Nyctotherus* and *Clevelandella*).

## References

[1] Affa’a F, Hickey DA, Strüder-Kypke M, Lynn DH. 2004 Phylogenetic position of species in the genera *Anoplophrya*, *Plagiotoma*, and *Nyctotheroides* (Phylum Ciliophora), endosymbiotic ciliates of annelids and anurans. Journal of Eukaryotic Microbiology, 51(3), 301–306. 15218698 10.1111/j.1550-7408.2004.tb00570.x

[2] Albaret JL. 1975 Étude systématique et cytologique sur les ciliés hétérotriches endocommensaux. Mémoires du Muséum National d’Histoire Naturelle, 89, 1–114.

[3] Corliss JO. 1977 Annotated assignment of families and genera to the orders and classes currently comprising the Corlissian scheme of higher classification for the phylum Ciliophora. Transactions of the American Microscopical Society, 96(1), 104–140.

[4] Corliss JO. 1979 The ciliated protozoa: Characterization, classification, and guide of the literature, 2nd ed., London: Pergamon Press p. 297–298.

[5] da Silva Paiva T, do Nascimento Borges B, da Silva-Neto ID. 2013 Phylogenetic study of class Armophorea (Alveolata, Ciliophora) based on 18S-rDNA data. Genetics and Molecular Biology, 36(4), 571–585. 24385862 10.1590/S1415-47572013000400017 PMC3873190

[6] Darriba D, Taboada GL, Doallo R, Posada D. 2012 jModel Test 2: more models, new heuristics and parallel computing. Nature Methods, 9, 772. 10.1038/nmeth.2109 PMC4594756 22847109

[7] Earl PR. 1972 Synopsis of the Plagiotomoidea, new superfamily (Protozoa). Acta Protozoologica, 9, 247–261.

[8] Guindon S, Dufayard J-F, Lefort V, Anisimova M, Hordijk W, Gascuel O. 2010 New algorithms and methods to estimate Maximum-Likelihood phylogenies: assessing the performance of PhyML 3.0. Systematic Biology, 59(3), 307–321. 20525638 10.1093/sysbio/syq010

[9] Jankowski AW. 2007 Review of taxa. Guide to Zoology. Protists, Part 2, 534–546.

[10] Li LX, Wang JG, Xiao WH. 1998 Taxonomic studies of parasitic Nyctotherans from Chinese anura amphibians I. *Nyctotheroides*. Acta Hydrobiologica Sinica, 22(Suppl), 186–196.

[11] Li LX, Wang JG, Xiao WH. 2002 Taxonomic studies of parasitic Nyctotherans from Chinese anura amphibians IV. *Spirocytopharynxa* gen. nov. and *Macrocytopharynxa* gen. nov. Zoological Studies, 41(1), 77–84.

[12] Li M, Li C, Grim JN, Ponce-Gordo F, Wang G, Hong Z, Li W, Wu S. 2017 Supplemental description of *Nyctotheroides pyriformis* n. comb. (=*Macrocytopharynxa pyriformis* (Nie, 1932) Li et al. 2002) from frog hosts with consideration of the validity of the genus *Macrocytopharynxa* (Armophorea, Clevelandellida). European Journal of Protistology, 58, 152–163. 28314219 10.1016/j.ejop.2016.10.002

[13] Li M, Sun Z, Grim JN, Ponce-Gordo F, Wang GT, Zou H, Li W, Wu S. 2016 Morphology of *Nyctotheroides hubeiensis* Li et al. 1998 from frog hosts with molecular phylogenetic study of Clevelandellid ciliates (Armophorea, Clevelandellida). Journal of Eukaryotic Microbiology, 63(6), 751–759. 27096441 10.1111/jeu.12322

[14] Lynn DH. 2004 Morphology or molecules: how do we identify the major lineages of ciliates (Phylum Ciliophora). European Journal of Protistology, 39, 356–364.

[15] Lynn DH. 2008 The Ciliated Protozoa (3rd): Characterization, Classification, and Guide to the Literature. Springer: The Netherlands p. 364–366.

[16] Lynn DH, Wright AD. 2013 Biodiversity and molecular phylogeny of Australian *Clevelandella* species (Class Armophorea, Order Clevelandellida, Family Clevelandellidae), intestinal endosymbiotic ciliates in the wood-feeding roach *Panesthia cribrata* Saussure, 1864. Journal of Eukaryotic Microbiology, 60(4), 335–341. 23590673 10.1111/jeu.12037

[17] Medlin LK, Elwood HJ, Stickel S, Sogin ML. 1988 The characterization of enzymatically amplified eukaryotic 16S-like rRNA-coding regions. Gene, 71(2), 491–499. 3224833 10.1016/0378-1119(88)90066-2

[18] Nie D. 1932 On some intestinal ciliates from *Rana limnocharis* Gravenhorst. Biological Laboratory of the Science Society of China, 8(6), 183–199.

[19] de Puytorac P, Grain J. 1976 Ultrastructure du cortex buccal et évolution chez les ciliés. Protistologica, 12, 49–67.

[20] Ronquist F, Teslenko M, Mark PVD, Ayres DL, Darling A, Höhna S, Larget B, Liu L, Suchard MA, Huelsenbeck JP. 2012 MrBayes 3.2: efficient Bayesian phylogenetic inference and model choice across a large model space. Systematic Biology, 61(3), 539–542. 22357727 10.1093/sysbio/sys029 PMC3329765

[21] Sambrook J, Fritsch EF, Maniatis T. 1989 Analysis and cloning of eukaryotic genomic DNA, in Molecular Cloning: A Laboratory Manual. 2nd ed. Cold Spring Harbor Laboratory Press: New York p. 456–491.

[22] Tamura K, Stecher G, Peterson D, Filipski A, Kumar S. 2013 MEGA6: Molecular Evolutionary Genetics Analysis Version 6.0. Molecular Biology and Evolution, 30(12), 2725–2729. 24132122 10.1093/molbev/mst197 PMC3840312

[23] Wilbert N. 1975 Eine verbesserte Technik der Protargolimprägnation für Ciliaten. Mikrokosmos, 64, 171–179.

[24] Xiao WH, Wang JG, Li LX. 2002 Taxonomic studies of parasitic Nyctotherans from Chinese anura amphibians III. *Wichtermania*. Zoological Studies, 41(1), 69–76.

